# Predicting Antidiabetic Peptide Activity: A Machine Learning Perspective on Type 1 and Type 2 Diabetes

**DOI:** 10.3390/ijms251810020

**Published:** 2024-09-18

**Authors:** Kaida Cai, Zhe Zhang, Wenzhou Zhu, Xiangwei Liu, Tingqing Yu, Wang Liao

**Affiliations:** 1 Department of Epidemiology and Biostatistics, School of Public Health, Southeast University, Nanjing 210009, China; 2 Department of Statistics and Actuarial Science, School of Mathematics, Southeast University, Nanjing 211189, China; 213212053@seu.edu.cn (Z.Z.); 213210109@seu.edu.cn (W.Z.); 213210770@seu.edu.cn (X.L.); 3 Key Laboratory of Environmental Medicine Engineering, Ministry of Education, School of Public Health, Southeast University, Nanjing 210009, China; ytqing@seu.edu.cn; 4 Department of Nutrition and Food Hygiene, School of Public Health, Southeast University, Nanjing 210009, China

**Keywords:** diabetes, antidiabetic peptides, machine learning, feature selection, classification

## Abstract

Diabetes mellitus (DM) presents a critical global health challenge, characterized by persistent hyperglycemia and associated with substantial economic and health-related burdens. This study employs advanced machine-learning techniques to improve the prediction and classification of antidiabetic peptides, with a particular focus on differentiating those effective against T1DM from those targeting T2DM. We integrate feature selection with analysis methods, including logistic regression, support vector machines (SVM), and adaptive boosting (AdaBoost), to classify antidiabetic peptides based on key features. Feature selection through the Lasso-penalized method identifies critical peptide characteristics that significantly influence antidiabetic activity, thereby establishing a robust foundation for future peptide design. A comprehensive evaluation of logistic regression, SVM, and AdaBoost shows that AdaBoost consistently outperforms the other methods, making it the most effective approach for classifying antidiabetic peptides. This research underscores the potential of machine learning in the systematic evaluation of bioactive peptides, contributing to the advancement of peptide-based therapies for diabetes management.

## 1. Introduction

Diabetes mellitus (DM), a disorder marked by elevated blood glucose levels, has burgeoned into a pressing global health challenge. The International Diabetes Federation reported that, in 2021, approximately 537 million individuals were living with this disease, and this figure is anticipated to rise to 643 million by 2030 [1]. The DM pandemic has posed a significant social economic burden. It has been estimated that the global DM-related health expenditures reached USD 966 in 2021 and was projected to reach USD 1054 by 2045 [1]. In addition, the prolonged use of conventional drug treatments is associated with side-effects, such as gastrointestinal disorders, dizziness, and fatigue [2]. Thus, the development of cost-effective intervention strategies has become a focus of the related academic and industrial fields.

Oligopeptides released from food proteins via enzymatic hydrolysis or fermentations could exert physiologically regulatory functions in vivo, which are thus known as bioactive peptides [3]. A wide range of bioactivities of food protein-derived bioactive peptides have been reported, such as antioxidant activity, blood pressure regulation, and immune-modulatory and glycemic control [4]. Of note, several types of anti-diabetic peptides have been characterized based on their mechanisms of action, including α-glucosidase and α-amylase inhibitors, peptides inhibiting glucose absorption, insulinotropic peptides, and dipeptidyl peptidase IV (DPP-IV) inhibitors [5]. However, for most of these peptides, the in vivo effect was evaluated in animal models and humans with type 2 diabetes mellitus (T2DM) given the fact that T2DM accounts for more than 90% of DM cases [6]. Meanwhile, it has been reported that hydrolysate prepared from a number of cereal proteins could exert hypoglycemic effect in mice with type 1 diabetic mellitus (T1DM) [7], which indicates the presence of bioactive peptides targeting T1DM in food protein sources.

Activity-guided purifications have been widely applied as a pipeline used for bioactive peptide characterizations from food proteins, which is time-consuming. Herby, the delineations of structure and function relationship could be helpful in improving the screening efficiency of bioactive peptides. Some structural features of anti-diabetic peptides have been reported. For instance, peptides with proline (Pro) or alanine (Ala) residue at the second position of the N-terminal exhibit strong DPP-IV inhibitory activity [7]. The binding site of α-amylase by the α-amylase inhibitory peptides has been characterized through in silico structural modeling [8].

Machine learning, a subset of artificial intelligence, involves developing algorithms capable of learning from data and making predictions based on patterns within these datasets. In recent years, the application of machine learning in the biomedical field has shown significant promise, particularly in predicting the activity of bioactive peptides. For instance, various machine-learning methods have been developed to predict the activity of antihypertensive peptides, demonstrating the potential of these methods to analyze complex biological data and extract meaningful insights from peptide sequences [9,10,11]. In particular, deep-learning algorithms like long short-term memory (LSTM) networks have been increasingly utilized for studying the structure–activity relationships of bioactive peptides due to their effectiveness in handling sequential data. In our recent study, we successfully established an LSTM model for antihypertensive peptides, suggesting the feasibility of incorporating deep-learning algorithms for the structure–activity relationship studies of bioactive peptides [12,13]. Beyond deep learning, support vector machines (SVM) and adaptive boosting (AdaBoost) have also been applied in the context of bioactive peptide research [14]. SVM is known for its robustness in high-dimensional spaces and its ability to find the optimal hyperplane that maximizes the margin between different classes, making it a powerful tool for peptide classification tasks [15]. Similarly, AdaBoost, an ensemble learning technique, combines the outputs of multiple weak classifiers to create a strong classifier, thereby enhancing predictive performance [16]. Both SVM and AdaBoost have demonstrated potential in enhancing the accuracy and reliability of bioactive peptide predictions, particularly in distinguishing between peptides effective against different types of diabetes based on their activity profiles [17].

In the field of antidiabetic peptides, numerous studies have identified endogenous peptide sequences with the potential to target T1DM [18]. These findings lay a crucial groundwork for the development of predictive methods that can further leverage these sequences. Such methods are vital for screening peptides derived from food proteins, which may possess antidiabetic properties, thereby expanding their therapeutic applications beyond their natural biological roles [19]. Despite this potential, the majority of existing research has been limited to preliminary analyses. There is a notable lack of studies that systematically develop and rigorously compare machine-learning methods tailored specifically for this purpose, hindering progress in the field. Moreover, while machine-learning techniques have been applied to predict the activity of peptides in other contexts [9,10,20], such as antihypertensive peptides, there has been limited exploration of these methods in predicting antidiabetic peptides, particularly in distinguishing between peptides effective against different types of diabetes. This study aims to address these gaps by constructing machine-learning methods specifically for antidiabetic peptides and comparing the efficiency and precision of different machine-learning methods. The contribution of this study lies in its systematic approach to evaluating and optimizing machine-learning algorithms for peptide activity prediction, providing a robust framework that could guide future research in screening and identifying bioactive peptides for diabetes management. Additionally, this research offers new insights into the application of machine learning in bioactive peptide studies, advancing the field and potentially leading to more targeted and effective therapeutic interventions.

## 2. Results

### 2.1. Descriptive and Hypothesis Testing Analysis

**Figure 1:**
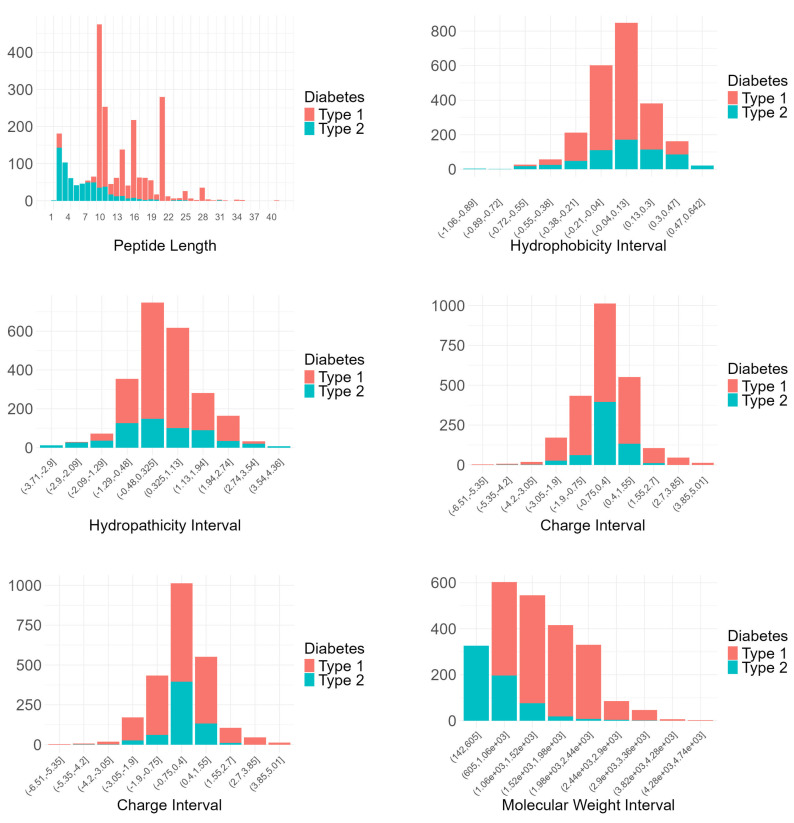
Bar plots of numeric features, including peptide length, hydrophobicity, hydropathicity, hydrophilicity, charge, and molecular weight.

**Figure 2:**
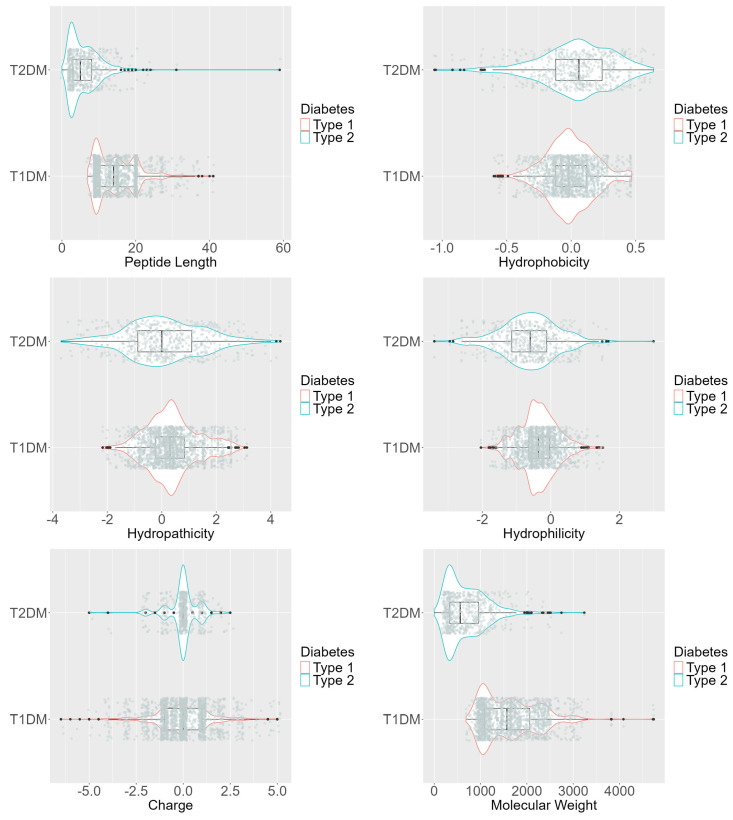
Violin plots of numeric features, including peptide length, hydrophobicity, hydropathicity, hydrophilicity, charge, and molecular weight.

To employ descriptive statistics for a preliminary exploration of the relationship between diabetes types and peptide features, including peptide length, hydrophobicity, hydropathicity, hydrophilicity, charge, and molecular weight, we construct bar plots and violin plots, as presented in Figure 1 and Figure 2. Based on the bar plots of Figure 1, some conclusions can be drawn regarding the distribution of diabetes types across various peptide characteristics. Shorter peptides (1–10) are more common, with significant presence for Type 1 diabetes at lengths 9 and 10. Hydrophobicity intervals reveal higher counts for Type 1 diabetes in the (−0.38, 0.3] range. Hydropathicity shows more Type 1 diabetes peptides in the (−1.29, 2.74] range, and hydrophilicity indicates higher counts for Type 1 in the (−1.84, 1.08] range. Charge intervals, particularly (−4.2 5.01], also show a higher count of Type 1 diabetes peptides. Molecular weight analysis reveals a dominance of Type 1 peptides in the (1000, 5000] range. These findings suggest that peptide characteristics like length, hydrophobicity, hydropathicity, hydrophilicity, charge, and molecular weight are more associated with peptides effective against Type 1 diabetes mellitus, highlighting their potential in antidiabetic peptide design.

The violin plots of Figure 2 illustrate several distinctions between peptides targeting T1DM and T2DM. Peptides associated with Type 1 diabetes generally exhibit a narrower range and lower values in terms of charge, molecular weight, hydropathicity, and hydrophobicity compared to those targeting Type 2 diabetes. Additionally, peptides for Type 1 diabetes tend to be shorter and more hydrophilic, whereas those for Type 2 diabetes are longer and less hydrophilic. These differences highlight the significance of these features in distinguishing peptides effective against the two types of diabetes.

**Figure 3:**
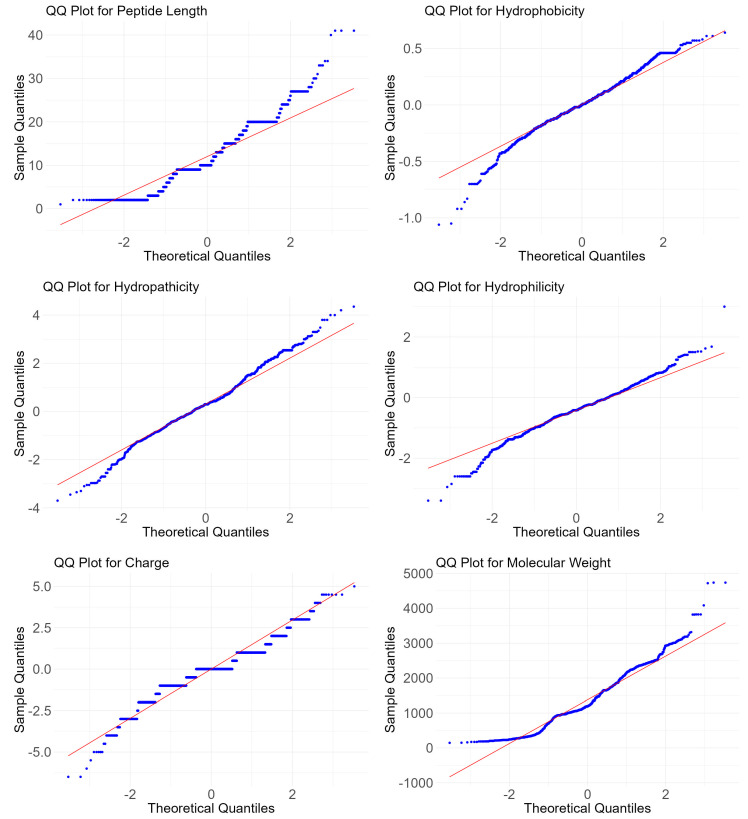
QQ plots of numeric features, including peptide length, hydrophobicity, hydropathicity, hydrophilicity, charge, and molecular weight.

To further explore the associations between these features and diabetes types, we conducted some hypothesis tests. Based on the QQ plots in Figure 3 and the results of the Shapiro–Wilk normality test in Table 1, some conclusions can be drawn regarding the distribution of the features. The Shapiro–Wilk test results indicate that none of the features, including peptide length, hydrophobicity, hydropathicity, hydrophilicity, charge, and molecular weight, follow a normal distribution, as evidenced by the very low *p*-values (all below $10^{-12}$). This non-normality is visually confirmed by the QQ plots, where the data points deviate significantly from the theoretical quantiles, especially at the tails of the distribution. Therefore, according to Shapiro and Wilk [21] and Wilcoxon [22], we use the Wilcoxon rank sum test, a non-parametric method, to assess the relationships between these features and diabetes types. The results of the Wilcoxon rank sum test, as shown in Table 1, further reveals significant differences in the distributions of these features between peptides effective against T1DM and T2DM. The low *p*-values from the Wilcoxon test (all below $10^{-03}$) suggest that these features are statistically distinct between the two types of peptides. Specifically, features such as peptide length and molecular weight show extremely low *p*-values (less than $2.200\times 10^{-16}$), highlighting their potential importance in differentiating the effectiveness of peptides against the two types of diabetes. These results emphasize the relevance of these peptide features in distinguishing between antidiabetic peptides targeting Type 1 and Type 2 diabetes mellitus, providing a foundational understanding for further analysis and method development.

### 2.2. Feature Selection

In the subsequent phase of our analysis, we employ the least absolute shrinkage and selection operator (Lasso) penalized method for feature selection, utilizing the glmnet package in R. The glmnet package automatically selects the optimal tuning parameter value to balance the trade-off between model fit and regularization. Through hyper-parameter tuning, the best parameter value, $6.724\times 10^{-5}$, is selected to prevent under-fitting. Lasso is particularly well-suited for handling high-dimensional data and promoting sparsity in the model by setting some coefficients to exactly zero. This approach effectively reduces the complexity of the model while highlighting the most relevant features. From the full set of features, Lasso ultimately selects 432 features deemed most predictive of antidiabetic peptide activity.

These selected features are categorized into four main groups: Amino Acid Composition, Sequence Characteristics, Physicochemical Properties, and Amino Acid Pair Counts, as summarized in Table 2. The Amino Acid Composition features count and position individual amino acids within the sequence, capturing the overall composition and distribution of the peptides. Sequence Characteristics include features like information entropy (Entropy), maximum consecutive repetitions of amino acids (MaxRepeat), Lempel–Ziv complexity (LZComplexity), and peptide length (PeptideLength), which reflect the complexity and structural properties of the peptides. Physicochemical Properties cover chemical attributes of the peptides, such as hydrophobicity, hydropathicity, hydrophilicity, charge, molecular weight, and toxin-related properties, all of which are crucial for distinguishing between peptides effective against Type 1 and Type 2 diabetes mellitus. Lastly, the Amino Acid Pair Counts provide detailed insights into local sequence patterns by counting specific amino acid pairs within the sequences.

The selection of these diverse features illustrates the multifaceted nature of peptide activity and underscores the necessity of considering a wide range of sequence characteristics for accurate classification. The integration of these features into the subsequent classification methods is expected to enhance their predictive accuracy and robustness in identifying antidiabetic peptides.

### 2.3. Performance Evaluation of Machine-Learning-Based Classification Methods

**Figure 4:**
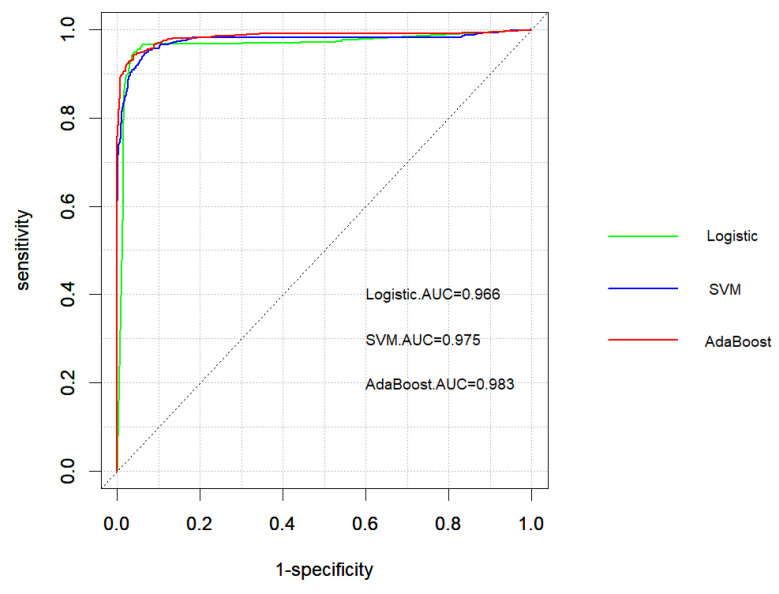
The ROC curves and AUC values of three classification methods.

The performance of the logistic regression, SVM, and AdaBoost classifiers are evaluated using the selected features. The receiver operating characteristic (ROC) curves for these classifiers are depicted in Figure 4. These curves illustrate the trade-off between sensitivity and specificity for each method, providing a visual representation of their discriminative ability. The ROC curves indicate that all three methods perform well in distinguishing between peptides effective against T1DM and T2DM, with AdaBoost demonstrating the highest discriminative power, followed closely by SVM and logistic regression. This demonstrates that AdaBoost is the most effective method among the three, achieving superior performance in terms of the area under the curve.

**Figure 5:**
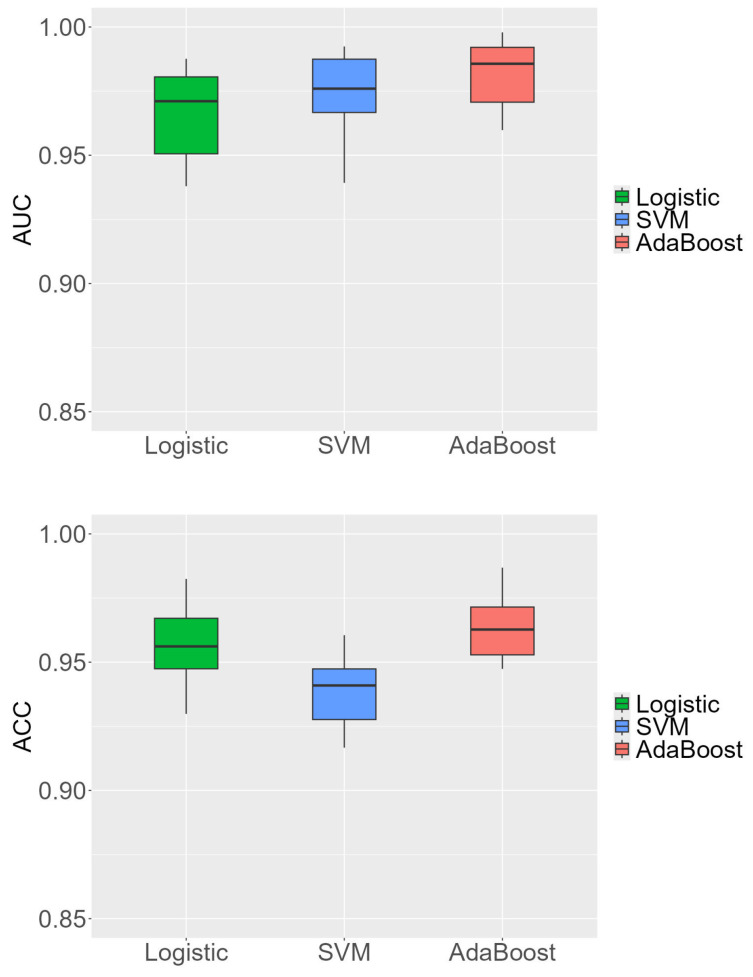
Boxplots of AUC and ACC values of three classification methods.

To comprehensively evaluate the performance of the logistic regression, SVM, and AdaBoost methods, we analyze their confusion matrices and various evaluation metrics. The confusion matrix for each method is presented in Table 3, while Table 4 summarizes the corresponding evaluation metrics, including the area under the curve (AUC), accurary (ACC), sensitivity, specificity, precision, F1 score, and Matthews correlation coefficient (MCC). Additionally, the boxplots in Figure 5 visualize the AUC and ACC values across different cross-validation folds.

The logistic regression method demonstrates a balanced performance, with an accuracy of 0.957 and an AUC of 0.966. The confusion matrix shows that it correctly classifies 1623 out of 1663 negative instances, resulting in a specificity of 0.933. It also correctly identifies 560 out of 618 positive instances, yielding a sensitivity of 0.965. The method has 40 false positives and 58 false negatives. The precision of the logistic regression method is 0.976, and its F1 score is 0.971. Moreover, its MCC is 0.890, further indicating that it has a balanced capability in handling both positive and negative classifications, even in the presence of potential class imbalance. These metrics indicate that the logistic regression method is reliable and effective in distinguishing between peptides effective against T1DM and T2DM. The boxplot in Figure 5 shows that the logistic regression method has a consistent AUC across different folds, with minimal variation.

The SVM method exhibits a high sensitivity of 0.990, correctly identifying 477 out of 494 positive instances. However, it has a higher number of false positives, with 123 misclassifications out of 1787 negative instances, leading to a lower specificity of 0.795. The overall accuracy of the SVM method is 0.939, and the AUC is 0.975. Despite its lower specificity, the SVM method’s precision is 0.931, and its F1 score is 0.960. The MCC of the SVM method is 0.839, reflecting its strong sensitivity but also acknowledging its trade-offs in terms of specificity. This high sensitivity makes SVM particularly effective at detecting true positives, which can be crucial in scenarios where correctly identifying positive instances is paramount. The boxplot indicates that the SVM method has a wider range of accuracy values across different folds, suggesting more variability in its performance. The AdaBoost method achieves the highest overall performance among the three methods, with an accuracy of 0.963 and an AUC of 0.983. It demonstrates a good balance between sensitivity (0.983) and specificity (0.905), correctly classifying 543 out of 571 positive instances and 1653 out of 1710 negative instances. The method has 57 false positives and 28 false negatives. The precision of the AdaBoost method is 0.967, and it has the highest F1 score of 0.975. Additionally, the MCC of 0.903 for AdaBoost further underscores its balanced and superior performance across both positive and negative classifications. These metrics highlight the robustness and reliability of the AdaBoost method in distinguishing between the two classes. The boxplot shows that AdaBoost has the highest median AUC, indicating superior performance across different folds.

Overall, AdaBoost clearly emerges as the most reliable and effective approach, offering a balanced and high level of performance across all metrics, including accuracy, sensitivity, specificity, F1 score, and MCC. Its ability to consistently outperform both logistic regression and SVM underscores its advantages in the classification of antidiabetic peptides. The results demonstrate that AdaBoost is the preferred choice for tasks requiring a high degree of precision, robustness, and reliability, as reflected by its superior MCC of 0.903. This further highlights its balanced performance across positive and negative classifications. The boxplots reinforce the robustness of AdaBoost by showing the highest median AUC and consistent accuracy across different cross-validation folds. These findings underscore the effectiveness of machine-learning methods, particularly AdaBoost, in classifying antidiabetic peptides and emphasize the importance of selecting the most appropriate method based on the specific requirements of the task.

## 3. Discussion

Although machine-learning algorithms have been widely applied to study the structure and function of bioactive peptides, including antihypertensive peptides [23], anticancer peptides [24], and antimicrobial peptides [25], their application in the research of antidiabetic peptides has seen significant progress recently. For instance, recent works, such as Yue et al. [26], have already explored the use of deep-learning models for predicting antidiabetic peptides. Their study utilized the BioDADPep database with a specific focus on peptides related to T1DM and T2DM, applying deep-learning techniques for peptide sequence generation and classification. In contrast, our study emphasizes a more comprehensive feature extraction process, analyzing peptide sequence characteristics such as length, hydrophobicity, and molecular weight. By integrating conventional statistical methods, like Lasso for feature selection, with machine-learning techniques such as SVM and AdaBoost, our approach prioritizes model interpretability. While Yue et al. [26]’s method demonstrated high accuracy with CNN models, our results indicate that AdaBoost consistently outperforms SVM and logistic regression across multiple metrics. The deep-learning models in their study offer valuable insights into generating potential ADPs, but the interpretability of these models is limited due to their black-box nature. In comparison, our study highlights the most relevant features for peptide activity prediction, providing a clearer understanding that can guide further research in bioactive peptide design and therapeutic applications. Both studies contribute valuable methodologies to antidiabetic peptide prediction. However, our work introduces a framework that balances accuracy and interpretability, making it a unique contribution to the field.

A key innovation of our study lies in the integration of machine learning with statistical feature selection methods, particularly the use of Lasso to ensure interpretability and robustness [27]. While machine-learning methods such as AdaBoost and SVM are established techniques, combining them with Lasso allows for the selection of the most predictive features, improving model interpretability and ensuring that the identified features are biologically meaningful [27,28]. Additionally, our comprehensive approach to feature extraction goes beyond the scope of existing studies by incorporating sequence characteristics like amino acid pair counts, information entropy, and Lempel–Ziv complexity, which enable a deeper understanding of peptide structure and function [29,30,31]. This unique combination of statistical and machine-learning methods allows us to differentiate between peptides effective against T1DM and T2DM, providing novel insights into bioactive peptide prediction. Our work advances the field by offering a methodology that balances predictive accuracy with interpretability, a contribution that is not fully explored in previous studies.

The findings from our analysis highlight the effectiveness of various machine-learning methods, particularly the AdaBoost method, in classifying antidiabetic peptides. Our approach of using descriptive statistics, hypothesis tests, and machine-learning methods provides a comprehensive evaluation of the distinguishing features between peptides effective against T1DM and T2DM, which could advance the precision intervention for DM. Although a recent study also reported a machine-learning predictor for the antidiabetic peptides by distinguishing the target DM type [19], the model did not consider the connection of each input feature with the output feature.

Our descriptive statistical analysis, through bar plots and violin plots, revealed distinct differences in peptide characteristics between the two diabetes types. Peptides associated with T2DM were generally longer, more hydrophobic, and had higher molecular weights compared to those targeting T1DM. These distinctions suggest that specific peptide features significantly influence their antidiabetic potential. The hypothesis tests further confirmed the non-normality of the peptide features and indicated significant differences between the distributions of these features for the two diabetes types. The Shapiro–Wilk normality test results demonstrated that none of the features followed a normal distribution, justifying our use of the non-parametric Wilcoxon rank sum test [21,22]. This test revealed statistically significant differences in all examined features, underscoring their relevance in distinguishing between the two types of antidiabetic peptides.

In the subsequent phase, feature selection using the Lasso method allowed us to identify the most predictive features, effectively reducing the model complexity while maintaining high predictive accuracy. The selected features included a wide range of peptide characteristics, such as amino acid counts, positions, and various physicochemical properties, highlighting the multifaceted nature of peptide bioactivity. As we demonstrated in the present study, the peptide length and molecular weight of the peptide have a strong connection with the its activity, suggesting the significance of the peptide sequence. Indeed, this notion has been implicated in our previous study that constructed an LSTM-based deep-learning model for predicting antihypertensive peptides [12]. Thus, we extracted multiple features of the peptide sequence and identified the most essential features, which was ignored by previous studies on constructing the predicting method for the antidiabetic peptides. Notably, the identifications of the essential features could also provide guidance for the antidiabetic peptide design.

The evaluation of the logistic regression, SVM, and AdaBoost methods revealed that, while all three approaches performed competently, AdaBoost consistently outshone the others in overall performance. The ROC curves and AUC values highlighted AdaBoost’s superior discriminative power, clearly surpassing both SVM and logistic regression. This superiority was further confirmed by the confusion matrices and evaluation metrics, with AdaBoost achieving the highest scores in accuracy, precision, F1, and MCC. The consistency of AdaBoost’s performance across different cross-validation folds, as illustrated by the boxplots, underscores its robustness and reliability. This consistent high performance suggests that AdaBoost is particularly well-suited for real-world applications requiring accurate and dependable classification. While other methods have their merits, the results of this study firmly establish AdaBoost as the most effective tool for classifying antidiabetic peptides, making it the preferred choice in scenarios where precision and reliability are paramount.

While our current study focuses on the binary classification of antidiabetic peptides as effective against either T1DM or T2DM, we recognize the potential for some peptides to exhibit dual efficacy. Given the limitations of our dataset, which lacks explicit labels for such peptides, we propose leveraging AdaBoost’s probabilistic outputs as a practical solution. AdaBoost not only provides binary classification but also generates probabilistic scores that reflect the likelihood of a peptide belonging to each class [16,32,33]. Peptides with balanced probabilities for both T1DM and T2DM could be flagged for further investigation as candidates with possible dual efficacy. This approach, while not directly addressing the absence of explicit data on dual efficacy, offers a way to explore the potential for peptides to influence both diabetes types without requiring additional data. The probabilistic interpretation enhances the flexibility of our model, allowing for a nuanced understanding of peptide behavior and supporting the identification of peptides that may warrant further experimental validation. This solution aligns with our broader aim of advancing predictive methods while ensuring interpretability and robustness in the classification of antidiabetic peptides.

## 4. Materials and Methods

### 4.1. Data Acquisition

The dataset utilized in this study is sourced from BioDADPep, a comprehensive bioinformatics database specifically curated for antidiabetic peptides [34]. From the processed data, seven key features are systematically extracted, which include peptide sequence, peptide length, hydrophobicity, hydropathicity, hydrophilicity, charge, and molecular weight [35,36,37]. These features are selected based on their relevance to the study’s objectives and their potential impact on the biological activity of the peptides. The response variable in this dataset is a binary indicator distinguishing between two types of diabetes: Type 1and Type 2 diabetes mellitus. It is encoded as 0 for peptides effective against T1DM and 1 for those targeting T2DM. After a meticulous process of removing instances with incomplete data, the final dataset comprised 2281 objects. To ensure a robust analysis, we use ten-fold cross-validation. This approach divides the dataset into ten subsets, with each subset being used as a test set once while the remaining nine subsets are used for training. This method provides a comprehensive and reliable assessment of the models’ effectiveness in predicting the type of diabetes mellitus.

### 4.2. Feature Extraction and Characterization of Peptide Sequences

In our study, we analyze peptide sequences to extract various features, integrating them with other features for use in classification methods to predict antidiabetic activity. Table 5 catalogs these extracted features, detailing their specific nature and role in the analysis. PairCounts quantifies the frequency of each possible amino acid pair combination, ranging from ‘AA’ to ‘ZZ’, within the sequence, providing comprehensive insights into local sequence patterns. AminoCounts measures the total occurrences of each amino acid, from ‘A’ to ‘Z’, reflecting the overall composition of the peptides. AvgPositions calculates the average position of each amino acid type within the sequences, offering a perspective on the distribution of amino acids throughout the sequence. Entropy assesses the information entropy of the sequences, which measures the randomness or diversity of amino acid arrangements, indicative of the sequence’s structural complexity. MaxRepeat identifies the maximum consecutive repetitions of any single amino acid, highlighting regions of potential repetitive functional motifs. LZComplexity evaluates the sequence’s complexity using the Lempel–Ziv algorithm, which provides a measure of the sequence’s overall structural intricacy. These features are meticulously selected and extracted to enrich the dataset, enhancing the predictive accuracy of our classification methods in identifying peptides with antidiabetic properties.

### 4.3. Machine-Learning-Based Methods for Classification

In this study, we apply three machine-learning methods, logistic regression, support vector machines, and adaptive boosting, to classify antidiabetic peptides [28,38,39,40]. We evaluate the effectiveness of these methods by comparing their performance metrics, such as the AUC, F1 score, precision, sensitivity, specificity, and MCC, with those of logistic regression, which serves as a baseline model. This comparison aims to highlight the strengths and potential advantages of each method in addressing the complex challenges of biomedical data classification.

The logistic regression model determines the probability that a particular input is assigned to the category marked as 1 rather than 0 [38,41]. This determination uses the logistic function, which is expressed as follows:

(1) $$\sigma \left(X\right)=\frac{1}{1+\exp\left(-(\beta_{0}+\beta_{1}X_{1}+\beta_{2}X_{2}+\cdots +\beta_{p}X_{p})\right)}$$

where σ(X)=P(Y=1|X) is the probability that the dependent variable *Y* equals 1 given the predictor variables *X*, exp represents the exponential function, and $\beta_{0},\beta_{1},\ldots ,\beta_{p}$ are the parameters of the model that need to be estimated. The coefficients are typically estimated using maximum likelihood estimation (MLE). This method optimizes the parameter values to maximize the likelihood that they would produce the observed sample. For the independent features, $x_{i}$, and the dependent features, $y_{i}$, in the dataset, and with $\beta ={\left(\beta_{0},\beta_{1},\ldots ,\beta_{p}\right)}^{⊤}$, the log-likelihood function is given by

(2) $$\ell \left(\beta \right)=\sum_{i=1}^{n}\left[y_{i}\log P\left(y_{i}=1|x_{i}\right)+\left(1-y_{i}\right)\log\left(1-P\left(y_{i}=1|x_{i}\right)\right)\right]$$

Support vector machines are supervised learning methods known for their robustness in classification tasks. SVM seeks to find a hyperplane in a high-dimensional space that best separates data points of different classes. The optimization problem in SVM involves maximizing the margin between classes while allowing some misclassifications, controlled by slack variables [15,40]. AdaBoost is an ensemble learning method that iteratively focuses on misclassified instances by adjusting their weights, thereby improving the overall model accuracy [16,39]. The model combines multiple weak classifiers to form a strong classifier, where each classifier’s influence is determined by its error rate. The final prediction is made by taking the weighted sum of all classifiers, emphasizing those with better performance.

### 4.4. Feature Selection Using the Lasso-Penalized Method

In logistic regression, the presence of many predictors or multicollinearity among them can lead to overfitting and instability in the coefficient estimates. Regularization techniques such as Lasso offer solutions to these issues by modifying the loss function to include a penalty term [27,42]. Employing Lasso in logistic regression allows for the effective handling of high-dimensional data by identifying significant predictors and excluding irrelevant ones, thereby increasing the robustness and explanatory power of the model.

Lasso applies an L1 penalty to the coefficients of the logistic regression model, which is expressed as

(3) $$L_{\mathrm{Lasso}}\left(\beta \right)=\ell \left(\beta \right)-\lambda \sum_{j=1}^{p}\left|\beta_{j}\right|$$

where ℓ(β) is the log-likelihood function of the logistic regression and λ is a non-negative tuning parameter controlling the strength of the penalty. This L1 penalty encourages sparsity in the model parameters, effectively performing feature selection by setting some coefficients to exactly zero when λ is sufficiently large.

### 4.5. Evaluation Metrics for Classification Performance

The efficacy of the classification models in this study is quantitatively assessed using several standard metrics derived from the confusion matrix, as depicted in Table 6. These metrics include the ROC curve, AUC, ACC, sensitivity, specificity, precision, F1 score, and MCC. Collectively, these metrics enable a comprehensive evaluation of model performance, ensuring that the models are assessed from various perspectives to capture all aspects of their predictive capabilities.

Accuracy is a metric used to evaluate the overall performance of a classification model. It is defined as the ratio of correctly predicted instances to the total instances in the dataset. Specifically, ACC is calculated as

(4) $$\mathrm{ACC}=\frac{\mathrm{TP}+\mathrm{TN}}{\mathrm{TP}+\mathrm{TN}+\mathrm{FP}+\mathrm{FN}}$$

where TP and TN are the instances correctly predicted by the model and FP and FN represent the misclassified instances. ACC provides a straightforward measure of how often the classifier is correct, but it may be misleading in cases of imbalanced datasets. The ROC curve is a graphical representation of a classification model’s ability to distinguish between positive and negative classes across various threshold settings. It plots the true positive rate (sensitivity) against the false positive rate (1—specificity) at different threshold levels. An ROC curve closer to the top left corner of the plot indicates better performance, as it represents higher sensitivity and lower false positive rates. The ROC curve provides a visual way to assess the trade-offs between sensitivity and specificity, and the AUC is a single scalar value summarizing the overall performance of the model. Typically, the AUC ranges from 0 to 1, where an AUC of 0.5 suggests no discriminative ability (equivalent to random guessing), and a value closer to 1 indicates a higher accuracy in classification. Precision (Pre) and Sensitivity (Sen) are calculated from the confusion matrix as follows:

(5) $$\mathrm{Pre}=\frac{\mathrm{TP}}{\mathrm{TP}+\mathrm{FP}}\;\mathrm{Sen}=\frac{\mathrm{TP}}{\mathrm{TP}+\mathrm{FN}}$$

where precision reflects the accuracy of positive predictions and sensitivity measures the proportion of actual positives correctly identified. Specificity (Sp), another key metric, indicates the proportion of actual negatives correctly identified and is defined as

(6) $$\mathrm{Sp}=\frac{\mathrm{TN}}{\mathrm{FP}+\mathrm{TN}}$$

The F1 score is the harmonic mean of precision and sensitivity, providing a balance between the two. It is especially useful when the class distribution is uneven. The F1 score is defined as

(7) $$F1=2\cdot \frac{\mathrm{Pre}\cdot \mathrm{Sen}}{\mathrm{Pre}+\mathrm{Sen}}$$

serving as a single metric that combines both the precision and recall of the model, where higher values suggest better model performance. Another important metric to consider is the Matthews correlation coefficient, which provides a balanced evaluation of classification performance, especially in cases of imbalanced datasets. MCC takes into account TP, TN, FP, and FN to produce a value between −1 and 1, where 1 indicates a perfect prediction, 0 represents no better than random guessing, and −1 signifies complete disagreement between predicted and actual classifications. The MCC is calculated as

(8) $$\mathrm{MCC}=\frac{\mathrm{TP}\cdot \mathrm{TN}-\mathrm{FP}\cdot \mathrm{FN}}{\sqrt{\left(\mathrm{TP}+\mathrm{FP})(\mathrm{TP}+\mathrm{FN})(\mathrm{TN}+\mathrm{FP})(\mathrm{TN}+\mathrm{FN}\right)}}$$

MCC is particularly useful for datasets with uneven class distributions, as it evaluates all four elements of the confusion matrix, providing a more reliable performance metric than accuracy in such cases.

## 5. Conclusions

Our findings underscore the importance of feature selection and method evaluation in developing accurate predictive methods for biomedical applications. By systematically analyzing peptide features and employing robust statistical and machine-learning methods, we identified key characteristics that distinguish peptides effective against T1DM and T2DM. The identified peptide features and their associations with antidiabetic activity provide valuable insights for future research and peptide design. Moreover, the AdaBoost method demonstrated the highest overall performance, indicating its potential as a reliable tool for peptide classification and its applicability in screening and identifying bioactive peptides with antidiabetic properties.

While our study has shown promising results, there are several important limitations to consider. Although the dataset we used is comprehensive, it may not fully capture the wide diversity of antidiabetic peptides, which could affect the generalizability of our models. Future research could focus on expanding the model to account for peptides that may be effective for both T1DM and T2DM, as more comprehensive datasets become available. Additionally, while our current model focuses on binary classification, we recognize the importance of considering peptide affinity and pharmacodynamic activity to differentiate between peptides with varying levels of potency. Incorporating affinity data into future models will allow for a more refined classification that distinguishes peptides with stronger and weaker effects. We also recognize the importance of validating the predicted activity and affinity of the peptides through in vivo experiments and the use of decoy peptides with a proven lack of activity as negative controls, which we will explore in future research. Moreover, while previous studies have applied deep-learning models for antidiabetic peptide prediction, future work could focus on further developing and refining machine-learning techniques to improve the ability to model complex peptide interactions and identify features that may enhance prediction accuracy.

## Figures and Tables

**Table 1:** *p*-values from the Shapiro–Wilk normality test and Wilcoxon rank sum test.

Feature	Shapiro–Wilk *p*-Value	Wilcoxon *p*-Value
Peptide length	<2.200 $\times \;10^{-16}$	<2.200 $\times \;10^{-16}$
Hydrophobicity	$3.234\times 10^{-14}$	$4.016\times 10^{-8}$
Hydropathicity	$7.603\times 10^{-12}$	$1.745\times 10^{-7}$
Hydrophilicity	$3.700\times 10^{-15}$	$3.330\times 10^{-14}$
Charge	<2.200 $\times \;10^{-16}$	2.406 $\times \;10^{-3}$
Molecular weight	<2.200 $\times \;10^{-16}$	<2.200 $\times \;10^{-16}$

**Table 2:** Summary of features selected by Lasso for predicting antidiabetic peptide activity.

Feature Category	Description and Examples of Selected Features
Amino Acid Composition	Counts and positions of individual amino acids (e.g., AminoCounts, AvgPositions)
Sequence Characteristics	Information entropy (Entropy), maximum consecutive repetitions (MaxRepeat), Lempel–Ziv complexity (LZComplexity), peptide length (PeptideLength)
Physicochemical Properties	Hydrophobicity, hydropathicity, hydrophilicity, charge, molecular weight, toxin-related properties
Amino Acid Pair Counts	Counts of specific amino acid pairs (e.g., PairCounts)
**Total Features Selected**	432

**Table 3:** Confusion matrix of three classification methods.

Actual\Predicted	Logistic		SVM		AdaBoost	
	0	1	0	1	0	1
0	1623	40	1664	123	1653	57
1	58	560	17	477	28	543

**Table 4:** Values of evaluation metrices of three classification methods.

Metrics	Logistic	SVM	Adaboost
ACC	0.957	0.939	0.963
AUC	0.966	0.975	0.983
Sensitivity	0.965	0.990	0.983
Specificity	0.933	0.795	0.905
Precision	0.976	0.931	0.967
F1 score	0.971	0.960	0.975
MCC	0.890	0.839	0.903

**Table 5:** Overview of features extracted from peptide sequences.

Feature Name	Description
PairCounts	Counts of each possible amino acid pair in the sequence
AminoCounts	Total count of each amino acid type in the sequence
AvgPositions	Average position of each amino acid within the sequence
Entropy	Information entropy of the amino acid sequence
MaxRepeat	Maximum consecutive repeats of any amino acid
LZComplexity	Lempel–Ziv complexity of the sequence

**Table 6:** Confusion matrix for evaluating classification model performance.

	Actual Positive	Actual Negative
**Predicted Positive**	True Positive (TP)	False Positive (FP)
**Predicted Negative**	False Negative (FN)	True Negative (TN)

## Data Availability

The data presented in the study are available in BioDADPep at http://omicsbase.com/BioDADPep (accessed on 18 October 2023).
